# High Vegetable Fats Intake Is Associated with High Resting Energy Expenditure in Vegetarians

**DOI:** 10.3390/nu7075259

**Published:** 2015-07-17

**Authors:** Tiziana Montalcini, Daniele De Bonis, Yvelise Ferro, Ilaria Carè, Elisa Mazza, Francesca Accattato, Marta Greco, Daniela Foti, Stefano Romeo, Elio Gulletta, Arturo Pujia

**Affiliations:** 1 Department of Clinical and Experimental Medicine, University Magna Grecia, Catanzaro 88100, Italy; 2 Department of Medical and Surgical Science, University Magna Grecia, Catanzaro 88100, Italy; E-Mails: daniele.db@gmail.com (D.D.B.); yferro@unicz.it (Y.F.); ilariacare@tiscali.it (I.C.); elisamazza@inwind.it (E.M.); romeo@unicz.it (S.R.); pujia@unicz.it (A.P.); 3 Department of Health Sciences, University Magna Grecia, Catanzaro 88100, Italy; E-Mails: francescaaccattato@libero.it (F.A.); mgreco2004@yahoo.it (M.G.); foti@unicz.it (D.F.); gulletta@unicz.it (E.G.); 4 Department of Molecular and Clinical Medicine, University of Gothenburg, Gothenburg SE-413 45, Sweden

**Keywords:** vegetarians, plant rich diet, energy expenditure, obesity

## Abstract

It has been demonstrated that a vegetarian diet may be effective in reducing body weight, however, the underlying mechanisms are not entirely clear. We investigated whether there is a difference in resting energy expenditure between 26 vegetarians and 26 non-vegetarians and the correlation between some nutritional factors and inflammatory markers with resting energy expenditure. In this cross-sectional study, vegetarians and non-vegetarians were matched by age, body mass index and gender. All underwent instrumental examinations to assess the difference in body composition, nutrient intake and resting energy expenditure. Biochemical analyses and 12 different cytokines and growth factors were measured as an index of inflammatory state. A higher resting energy expenditure was found in vegetarians than in non-vegetarians (*p* = 0.008). Furthermore, a higher energy from diet, fibre, vegetable fats intake and interleukin-β (IL-1β) was found between the groups. In the univariate and multivariable analysis, resting energy expenditure was associated with vegetarian diet, free-fat mass and vegetable fats (*p* < 0.001; Slope in statistic (B) = 4.8; β = 0.42). After adjustment for cytokines, log_10_ interleukin-10 (IL-10) still correlated with resting energy expenditure (*p* = 0.02). Resting energy expenditure was positively correlated with a specific component of the vegetarian’s diet, *i.e.*, vegetable fats. Furthermore, we showed that IL-10 was positively associated with resting energy expenditure in this population.

## 1. Introduction

Obesity increases the risk of several chronic diseases like non-alcoholic fatty liver disease and asthma, but its biggest effect is probably on diabetes and cardiovascular disease [1,2,3]. At the moment, an effective strategy to reduce obesity incidence is lacking, thus obesity will consume an ever increasing proportion of national healthcare resources. Recently, it has been suggested that the prescription of vegetarian/vegan diets may be successful in reducing body weight [4,5]. Two mechanisms seem to account for this effect—the increased thermic effect of food and the reduced energy intake—probably both as a consequence of the low-fat content in the diet [6,7,8]. However, a study suggests that long-term adaptation to a vegetarian diet does not provide an enhanced thermic response, rather the thermic effect of food in vegetarians was 25% lower than in omnivores [9]. It has also been demonstrated that vegetarians have a higher resting metabolic rate than omnivores and that this effect may be mediated by some dietary components of a vegetarian diet [10,11]. Further investigations are needed to fully elucidate the underline mechanisms leading to a greater weight loss in vegetarian/vegan individuals compared with omnivores and to recommend this type of dietary intervention to prevent weight gain in the general population. In particular, the relationship between some common dietary components and resting energy expenditure (REE) needs to be determined. Since dietary patterns characterized by high intakes of red meat and high-fat dairy products have shown a relationship with both excess body weight and high cytokines levels [12], the influence of inflammation on REE needs also to be explored. In this regard, it has been demonstrated that some dietary patterns can have a significant impact on inflammatory markers [13,14]. Observational research has shown that vegetarians have lower levels of acute phase proteins than non-vegetarians [15]. Furthermore, some investigations have demonstrated the potential anti-inflammatory activities of various plant-based diets [16]. However, studies measuring the objective difference in cytokines between vegetarians and non-vegetarians and their influence on REE are lacking, and it may be possible that cytokines play a role in influencing REE. Furthermore, vegetarian dietary patterns may vary in different geographical regions. Consequently, we sought to investigate whether a difference in REE exists between vegetarians and non-vegetarians and which nutritional factors and cytokines correlate with REE.

## 2. Methods

In this cross-sectional study, we recruited 52 individuals: 26 self-report vegetarians and 26 non-vegetarians. They were matched by age, body mass index (BMI) and gender, and invited by newspapers ads to participate in the study which was conducted from June 2014 to January 2015 at the “Mater Domini” University Hospital. Of the vegetarians, we excluded individuals who had been following this diet for less than three years. We also excluded those from the study who, during the medical interview and examination, had clinical evidence of debilitating diseases, such as chronic illness (cancer, severe renal failure, sever liver insufficiency and chronic obstructive pulmonary disease), thyroid dysfunction and established cardiovascular disease (myocardial infarction, stroke, peripheral artery disease assessed), as well as subjects taking anti-obesity medications, psychotropic drugs, chronotropic agents and antioxidant supplements and vitamins. We also collected all the known classical cardiovascular (CVD) risk factors and information about current time spent in vigorous activity per week, consistent with the method used by Armstrong *et al*. [17]. The following criteria were used to define the distinct cardio-metabolic risk factors; diabetes: fasting blood glucose ≥ 126 mg/dL or antidiabetic treatment; hyperlipidemia: total cholesterol > 200 mg/dL and/or triglycerides > 200 mg/dL or lipid lowering drugs use; hypertension: systolic blood pressure ≥ 140 mmHg and/or diastolic blood pressure ≥ 90 mmHg or antihypertensive treatment; overweight: 25 kg/m^2^ ≤ BMI < 30 kg/m^2^; obesity: BMI ≥ 30 kg/m^2^; smoking: current smokers [18].

**Figure 1:**
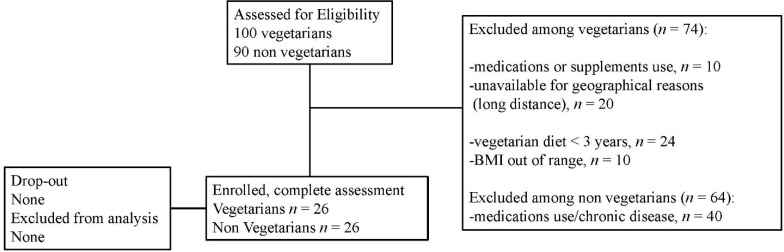
Flow diagram of participants. BMI, body mass index.

Written informed consent was obtained (flow chart of the population in Figure 1).

The protocol was approved by local ethical committee at the “Mater Domini” Azienda University Hospital (2013.1/CE). The investigation conforms to the principles outlined in the Declaration of Helsinki.

### 2.1. Blood Pressure Measurement

The measurement of the systemic blood pressure (BP) of both arms was obtained by a mercury sphygmomanometer (systolic blood pressure (SBP) and diastolic blood pressure (DBP)) as previously described [19]. Clinic BP was obtained in supine patients, after 5 min of quiet rest. A minimum of three BP readings were taken using an appropriate BP cuff size (the inflatable part of the BP cuff covered about 80 percent of the circumference of the upper arm).

### 2.2. Nutritional Intake

Seven-day diet records were obtained for dietary assessment and the participant’s nutritional intake was calculated using the nutritional software MetaDieta 3.0.1 (Metedasrl, San Benedetto del Tronto, Italy). Vegetarians were considered persons who do not consume animal products. However, those consumed eggs and dairy products were also included among vegetarians.

### 2.3. Anthropometric Measurements

All tests were performed after a 12 h overnight fasting. Before tests, participants had no caffeinated beverages between their evening meal and the conclusion of the tests on the examination’s morning. Body weight was measured before breakfast with the subjects lightly dressed, subtracting the weight of clothes. Body weight was measured with a calibrated scale and height measured with a wall-mounted stadiometer. BMI was calculated with the following equation: weight (kg)/height (m)^2^. Waist circumferences and hip circumferences (WC and HC) were measured with a nonstretchable tape over the unclothed abdomen at the narrowest point between the costal margin and iliac crest and over light clothing at the level of the widest diameter around the buttocks, respectively, as described in the past [20].

The handgrip strength was measured by dieticians previously trained in the technique. The handgrip strength was measured using a hydraulic hand dynamometer (Hersteller/manufactures; SAEHAN Corporation, Masan-Korea; Distributor Rehaforum Medical GmbH, Elmshorn-Germany) having less than 10% variation in results for various grip positions. Subjects were seated, with their elbows flexed at 90° and supported at the time of the measurement. Dieticians collected three measurements from each hand, and we used the mean value in all analyses. During the measurement, dieticians asked the subject to grip the dynamometer with maximum strength, and to hold the grip for at least three seconds. Handgrip strength is registered as maximum kilograms of strength applied during the registration [21].

Skin fold thickness was measured at the triceps (TSF) with the GIMA Skinfold Caliper (Gessate, Milan, Italy) [22].The site was measured three times, and the mean was calculated.

### 2.4. REE and Fuel Utilization Assessment

Fasting respiratory quotient (RQ, index of fuel utilisation) and the Resting Energy Expenditure (REE) were measured with the participants in their post-absorptive state in a sedentary position. Respiratory gas exchange was measured by Indirect Calorimetry using the open circuit technique between the hours of 7 a.m. and 8:30 a.m. after 48 h abstention from exercise. The Indirect Calorimetry instrument (Viasys Healthcare, Hoechberg, Germany) was used for all measurements. The participant rested quietly for 30 min in an isolated room with temperature controlled (21–24 °C) environment. The subject was then placed in a ventilated hood for at least 30 min, until steady state was achieved. Criteria for a valid measurement was a minimum of 15 min of steady state, with steady state determined as less than 10% fluctuation in minute ventilation and oxygen consumption and less than 5% fluctuation in RQ. RQ was calculated as CO_2_ production/O_2_ consumption. The interassay coefficient of variation (CV) is 6.7% [23].

### 2.5. Biochemical Evaluation

Routine analyses, including fasting glucose, total cholesterol, high-density lipoprotein (HDL), triglycerides and uric acid were obtained from fresh samples, whereas aliquots of serum or citrated plasma were frozen at −80 °C for subsequent laboratory determinations (cytokines).

Blood glucose, total cholesterol, HDL and triglycerides were performed by Cobas 6000 (Roche, Basel, Switzerland) using the commercially available Enzyme-Linked Immunosorbent Assay (ELISA) assay kits. We use fasting lipid levels to calculate the value for low density lipoprotein (LDL) cholesterol (Friedewald formula); all the above-mentioned assays were carried out according to the manufacturer’s instructions. Quality control was assessed daily for all determinations.

### 2.6. Measurement of Serum Cytokines

The serum concentrations of 12 different cytokines and growth factors (interleukin-1α (IL-1α), interleukin-1β (IL-1β), interleukin-2 (IL-2), interleukin-4 (IL-4), interleukin-6 (IL-6), interleukin-8 (IL-8), interleukin-10 (IL-10), Interferon-γ (IFN-γ), tumor necrosis factor α (TNF-α), monocyte chemotactic protein-1 (MCP-1), vascular endothelial growth factor (VEGF), and epidermal growth factor (EGF)) were simultaneously determined using the biochip analyser Evidence Investigator (Randox Labs, Antrim, UK) and the “Cytokine Array I and High sensitivity” kit (Randox, Antrim, UK), according to the manufacturer’s instructions. Briefly, the principle of this multianalyte testing relies on a sandwich ELISA, in which the analytes of interest are captured by specific antibodies bound to discrete regions of the surface chemistry of the biochip; horseradish peroxidase (HRP) labeled secondary antibodies, which specifically recognize the analytes, trigger a luminol-based electrochemiluminescent signal emission, registered by a charge-coupled device (CCD) camera and quantified by a software.

### 2.7. Air-Displacement Plethysmography (BOD POD)

Whole body air-displacement was evaluated with the BOD POD (Body Composition Tracking System, mode 2007A, Cosmed USA Inc., Concord, CA, USA). The BOD POD is a single fiberglass unit composed of two chambers. Each subject was tested in a one-piece swimsuit with a swim cap. Participants were also asked to void their bladder to minimize potential error due to excess water volume. Thoracic gas volume (TGV) was measured in all subjects and BOD POD conditions (*i.e.*, BOD POD_BH_ and BOD POD_FH_) according to the procedures described in the manual, total body fat was determined by the Siri equation [24,25]. Coefficient of variation (CV) was <3%.

### 2.8. Statistical Analysis

Data are reported as mean ± standard deviation (SD). Nineteen subjects in each group are required to detect a significant difference of REE equal to 15% with 80% power on a two-sided level of significance of 0.05.

Each quantitative trait was tested for normality using the Shapiro-Wilk normality test and, when required, it has been log-transformed.

A chi-square test was performed to compare the prevalence of the risk factors and a *t*-test was performed to compare the means between subjects with and without obesity.

The Pearson correlation was used to identify the variables correlated with the REE. Stepwise multivariable linear regression analysis was used to test the association between REE and the confounding variables selected among all that in the univariate analysis were associated with REE having a *p* < 0.1. Since energy from diet, plant protein and vegetable fats may be closely linked, to avoid one disappearing over the other in the regression due to this, in the multivariable linear regression analysis we performed two models. In the first Model (Model I), only free-fat mass, vegetarian diet and nutrients intake (*i.e.*, plant protein, vegetable fats and fiber) were included as dependent variables. In the second Model (Model II), only free-fat mass, vegetarian diet and energy from diet were included as dependent variables. Furthermore, we performed a third Model (Model III) to adjust for log transformed cytokines including also, as dependent variables, free-fat mass and vegetarian diet. The general linear model (GLM) was used to adjust REE for the time spent to exercise, age and gender. Significant differences were assumed to be present at *p* < 0.05 (two-tailed). All comparisons were performed using SPSS 20.0 for Windows (IBM Corporation, New York, NY, USA).

## 3. Results

All participants completed the study and all measurements were available for statistical analysis. There were 13 females in each group. The demographic characteristics and cardiovascular risk factor prevalence of the population are indicated in Table 1. A higher REE was found in vegetarians than in non-vegetarians (*p* = 0.01, Table 1). REE was higher in vegetarians than in non-vegetarians also after adjustment for free-fat mass (FFM) (Table 1).

**Table 1:** General, anthropometric and dietary intake characteristics among 26 vegetarian and 26 non-vegetarians.

Variables	Non-vegetarians (Mean ± SD)	Vegetarians (Mean ± SD)	*p*
Age (years)	30.5 ± 6.7	32.6 ± 8.4	0.33
Body weight (kg)	62.5 ± 9	62.5 ± 9	0.97
BMI (kg/m²)	21.82 ± 2	21.93 ± 2	0.88
WHR	0.80 ± 0.09	0.82 ± 0.07	0.48
WC (cm)	15.65 ± 1.4	16.34 ± 1.2	0.06
HC (cm)	33.59 ± 7.6	34.69 ± 2.8	0.49
Hand Grip (kg)	38.1 ± 12	34.96 ± 10	0.33
Estimated vigorous activity (MET·min·week^−1^)	8.2 ± 4	12.2 ± 4	0.02
SBP (mmHg)	114 ± 13	111 ± 12	0.48
DBP(mmHg)	72 ± 9	71 ± 9	0.70
Pulse pressure (mmHg)	42 ± 9	40 ± 8	0.57
HR (b/m)	67 ± 8	68 ± 8	0.73
***BOD POD assessment***			
Total body Fat (Kg)	14.6 ± 6	13.8 ± 8	0.68
Free-Fat Mass (Kg)	48.2 ± 9.6	49.1 ± 10.0	0.73
Total body fat (%)	23.4 ± 8	21.8 ±11	0.58
Free-Fat Mass (%)	76.5 ± 8	78.1 ± 11	0.58
***Indirect calorimetry assessment***			
REE (Kcal)	1268 ± 191	1473 ± 343	0.01
REE (Kcal) age, gender, exercise * adjusted	1313 ± 65	1603 ± 70	<0.001
REE (Kcal) FFM adjusted	1277 ± 152	1463 ± 244	0.02
REE (Kcal) FFM, age, gender, exercise adjusted	1254 ± 56	1536 ± 61	0.04
RQ	0.95 ± 0.11	0.87 ± 0.10	<0.001
***Dietary assessment***			
Energy intake (Kcal)	1866 ± 441	2118 ± 554	0.07
Proteins (g)	81 ± 32	67 ± 21	0.08
Carbohydrates (g)	237 ± 64	293 ± 91	0.01
Fats (g)	68 ± 22	83 ± 27	0.03
Animal Protein (g)	53 ± 28	13 ± 9	<0.001
Vegetable protein (g)	26 ± 8	53± 26	<0.001
Animal fats (g)	26 ± 11	14 ± 11	<0.001
Vegetable fats (g)	40 ± 15	68 ± 26	<0.001
fiber (g)	22 ± 6	37 ± 17	<0.001
Cholesterol (mg)	197 ± 101	87 ± 79	<0.001
***Risk factors prevalence***			
Smokers (%)	0.07(2)	0.23 (6)	0.30
Diabetes/hypertension (%)	0	0	0
Hypercholesterolemia (%)	0% (0)	0.38 (1)	0.31

Legend: BMI, body mass index; WHR, waist to hip ratio; WC, Waist circumferences; HC, hip circumferences; MET, metabolic equivalent of task; SBP, systolic blood pressure; DBP, diastolic blood pressure; HR, heart rate; REE, resting energy expenditure; FFM, free fat mass; RQ, respiratory quotient; BOD POD, Air-Displacement Plethysmography; * time spent for physical exercise.

Furthermore, a higher energy from diet, fibre, plant proteins and plant fats intake was found in vegetarians than in non-vegetarians (Table 1). When all cytokines were log_10_ transformed, only a difference in IL-1β was found between the two groups (higher in vegetarians than in non-vegetarians, *p* = 0.004, Table 2).

**Table 2:** Biochemical characteristics among 26 vegetarian and 26 non-vegetarians.

Variables	Non-vegetarians (Mean ± SD)	Vegetarians (Mean ± SD)	*p*
Glycemia, mg/dL (mmol/L)	85 ± 8 (4.72 ± 0.4)	86 ± 5 (4.7 ± 0.2)	0.47
Total cholesterol, mg/dL (mmol/L)	173 ± 28 (4.47 ± 0.7)	170 ± 33 (4.39 ± 0.8)	0.74
HDL-cholesterol, mg/dL (mmol/L)	64 ± 16 (1.65 ± 0.4)	61 ± 17 (1.58 ± 0.4)	0.51
LDL-cholesterol, mg/dL (mmol/L)	93 ± 27 (2.4 ± 0.7)	95 ± 25 (2.45 ± 0.6)	0.73
Triglycerides, mg/dL (mmol/L)	85 ± 67 (0.96±0.7)	71 ± 30 (0.8±0.3)	0.36
Uric acid (mg/dL)	4.7 ± 1.4	4.1 ± 1.0	0.13
***Cytokine evaluation***			
IL-2 (pg/mL)	2.42 ± 9.9 (0.0–46.3)	0.29 ± 1.4 (0.0–6.6)	0.32
IL-4 (pg/mL)	0.88 ± 1.3 (0.0–3.95)	0.97 ± 1.1 (0.0–2.75)	0.80
IL-6 (pg/mL)	1.52 ± 1.4 (0.0–6.6)	1.97 ± 2.8 (0.0–14)	0.50
IL-8 (pg/mL)	14.76 ± 12.2 (2.1–56)	14.60 ± 9.8 (2.1–43.1)	0.96
IL-10 (pg/mL)	0.53 ± 0.80 (0.0–2.1)	1.02 ± 1.08 (0.0–3.3)	0.08
VEGF (pg/mL)	234.75 ± 109 (69.6–492)	230.46 ± 126 (59.9–590)	0.90
INF γ (pg/mL)	0.12 ± 0.5 (0.0–2.7) ^§^	0	0.32
TNFα (pg/mL)	2.42 ± 1.1 (0.0–4.6)	2.41 ± 0.9 (0.0–4.4)	0.97
IL-1α (pg/mL)	0.21 ± 0.4 (0.0–1.8 )	0.18 ± 0.4 (0.0–2.1)	0.79
IL-1β (pg/mL)	0.31 ± 0.8 (0.0–2.5)	0.50 ± 0.8 (0.0–2.2)	0.46 0.04 *
***Cytokine evaluation***			
MCP-1 (pg/mL)	320.9 ± 133.7 (139–659)	376.6 ± 138.2 (62–615)	0.17
EGF (pg/mL)	122.01 ± 63.2 (18–263)	117.08 ± 55.6 (39–225)	0.78

Legend: T Cholesterol, total cholesterol; HDL, high density lipoprotein; LDL, low density lipoprotein; * log_10_ transformed; IL-1α, interleukin-1α; IL-1β, interleukin-1β; IL-2, interleukin-2; IL-4, interleukin-4; IL-6, interleukin-6; IL-8, interleukin-8; IL-10, interleukin-10; IFN-γ, Interferon-γ; TNF-α, tumor necrosis factor α; MCP-1, monocyte chemotactic protein-1; VEGF, vascular endothelial growth factor; EGF, epidermal growth factor; § min and max.

**Table 3:** Univariate analysis, Pearson correlation variables correlated with resting energy expenditure (REE).

Variables		Age	Gender	Free fat mass	Vegetarian diet	Energy Intake	Fiber	Animal protein	Vegetable fats	IL-6	IL-10	IL-1β
REE	*r*	0.39	0.52	0.65	0.35	0.42	0.505	0.41	0.613	0.36	0.58	−0.72
	*p*	0.041	0.005	<0.001	0.010	0.002	<0.001	0.002	<0.001	0.023	0.009	0.03

IL-6, interleukin-6; IL-10, interleukin-10; IL-1β, interleukin-1β.

**Table 4:** Multivariate analysis, factors associated with resting energy expenditure (REE) as dependent variable.

Dependent variable REE	B	SE	β	*p*
I Model *				
Fat-Free Mass	14.74	3.01	0.48	<0.001
Vegetable fats	4.88	1.14	0.42	<0.001
II Model **				
Fat-Free Mass	19.17	2.96	0.63	<0.001
Vegetarian diet	186.73	57.20	0.32	0.002
III Model ***				
Log_10_ IL-10	1474.23	60.92	0.86	0.002
Vegetarian diet	170.74	15.70	0.38	0.008

* I model, excluded variables: fiber, vegetal proteins, vegetarian diet; ** II model, excluded variables: Energy from diet; *** III model, excluded variables: Fat-Free Mass, interleukin-6 (IL-6), and interleukin-1β (IL-1β); B, Slope in statistic; SE, standard deviation.

Table 3 shows the factors significantly associated with REE (which were also associated with REE adjusted for FFM, data not shown) in the univariate analysis. Since vegetarians spent more time doing vigorous physical exercise every week than non-vegetarians (*p* = 0.021, Table 1), GLM was performed to adjust REE for physical exercise, as well as for age and gender, confirming the higher REE in vegetarians than in non-vegetarians (*p* = 0.008 after adjustment, value included also in Table 1). In the multivariable analysis (Table 4), REE (adjusted) was associated, as expected, with free-fat mass and, in Model I, with vegetable fats, and in Model II with a vegetarian diet, while in Model III, free-fat mass disappeared and only log_10_ IL-10 and vegetarian status remained correlated with REE (Table 4) (Vegetarian status and vegetable fats were correlated: *r* = 0.55 and *p* < 0.001).

## 4. Discussion

The main finding in our investigation was the significant difference in REE between vegetarians and non-vegetarians. In particular, a higher REE was found in vegetarians than in non-vegetarians, matched for BMI, and the significant difference also remained after an adjustment for physical exercise, age, gender and FFM (Table 1). We also found a greater post-absorptive fat utilisation (low RQ) in vegetarians than in non-vegetarians. Furthermore, we found lower cholesterol, higher plant proteins, higher plant fats and higher fibre intake in vegetarians than in non-vegetarians.

In our investigation, REE and post-absorptive fat utilisation did not correlate, while, as expected, REE was positively correlated with vegetarian status, free-fat mass, vegetable fats, energy from diet and some interleukins such as IL-6 and IL-10, and negatively, with animal proteins and IL-1β. However, after adjustments for confounders in the multivariable analysis, REE continued to be associated with a vegetarian diet, free-fat mass, vegetable fats and IL-10 only (Table 4). In addition, in our investigation, a vegetarian diet and vegetable fats were positively correlated (see the Results Section).

This finding is very interesting and original since we demonstrated that there may be specific dietary components in vegetarians, *i.e.*, vegetable fats which, even in association with other factors (such as free-fat mass), may contribute to an increased REE.

In addition, in this population we demonstrated that IL-10 may be an important inflammatory mediator positively associated with REE.

Our results can be considered in line with findings by other authors suggesting that dietary macronutrient composition may influence resting metabolic rate [10]. In this regard, vegetable fats may have a role.

In fact, despite the numerous reported benefits of exercise, many studies failed to show significant advantages of exercise in terms of changes in REE or body composition [26,27,28].

Conversely, it has been shown that habitual high-fat consumers have a significantly higher REE than low-fat consumers [29] thus*,* habitual consumption of a high vegetal-fat diet in vegetarians may lead to physiological adaptations in the form of increased REE independent of exercise.

In recent intervention trials, free-living individuals added nuts, rich in vegetable fat, to their habitual diet without weight gain despite the increased energy intake [30,31,32]. Routine consumption of nuts increases REE [33,34]. In rural China, where obesity is uncommon, a vegetarian diet was found to be associated with greater energy intake [11], as in our investigation. So, although not definitively recognised, vegetable fats may have a role in increasing REE.

Studies investigating the thermic effect of food were inconclusive [6,9]. It has been shown that in low-protein fed animals increased thermogenesis was due not only to greater brown adipose tissue metabolism but also to greater voluntary physical activity [35] suggesting that increased REE may be attributable to other factors rather than thermogenesis [11]. However, we did not assess this aspect in our investigation.

The original finding of our study is the association between plasma IL-10 and REE. IL-10, secreted by activated monocytes/macrophages and T-cells, is a key anti-inflammatory cytokine that promotes inflammatory resolution by blocking signals that initiate the synthesis of pro-inflammatory proteins [36]. Habitual exercise was associated with high IL-10 levels to constrain increases in inflammatory status in response to acute stress [37].

Of interest, it has been demonstrated that exercise increases hypothalamic levels of IL-10 improving insulin and leptin action and that IL-10 suppresses hyperphagia in rodents [38].

However, in our investigation, the cytokines’ assessment was performed at rest, after at least 48 h abstention from exercise. In fact, all cytokine concentrations were low in both vegetarians and non-vegetarians if compared with the much more exaggerated inflammatory cytokine response occurring during or immediately after exercise [39]. It is well known that inflammatory responses may be modified by dietary components, especially lipids [40,41,42]. High levels of IL-10 have been observed in subjects with a high adherence to a Mediterranean diet or who consume antioxidant-rich food [43,44]. Our finding, therefore, suggests a role for IL-10 in controlling food intake and REE independent of exercise that deserves further exploration, also in light of the fact that IL-10 profoundly reduced lipid accumulation in the vessel wall, preventing atherosclerosis [45,46,47].

In our study, we assessed pro-inflammatory and anti-inflammatory cytokines that were not examined together in previous studies. In fact, the study of Paalani *et al*. [15] only analyzed inflammatory markers that included pro-inflammatory markers such as IL-6 and TNF-α and anti-inflammatory cytokines such as IL-10 as well as an acute phase protein (CRP). They demonstrated that, after controlling for all other covariates, a vegetarian diet was associated with lower CRP levels while exercise frequency was associated with higher IL-10 levels. However, the objective of the study was to investigate the diverse determinants of ethnic-specific differences in inflammation and how inflammation levels were influenced by lifestyle behaviors while our study focused mainly on the specific difference in inflammation markers between vegetarians and non-vegetarians and the relationship between inflammation markers and REE. Our investigation was based on the observation that some dietary patterns have shown a relationship with both BMI and inflammation [12], thus the influence of some cytokines on REE needs to be explored. 

In our study, some strengths and weaknesses must be addressed. It is difficult to discern whether the advantages in terms of energy balance attributed to vegetarians could be generalised to all vegetarians or vegans or even to moderate meat-eaters following a healthy diet. Due to the study design, this investigation did not address diet sustainability in the longer term. Furthermore, our study was limited by its small size and cross-sectional design. However, the statistical analysis is robust and adequate. Furthermore, a recent investigation on this topic enrolled a number of participants for a group similar to ours [48].

Our results were not purely random as established by previous investigations [10,11] and were confirmed by multiple statistical analyses. The investigation was carried out on representative samples of young Italians with a Mediterranean diet, potentially increasing knowledge of this issue from a geographical perspective. However, this fact may have affected our results and may explain why we did not find any significant differences in blood pressure and glucose between vegetarians and non-vegetarians. In fact, vegans/vegetarians were found to have lower blood pressure and glucose concentrations than omnivores [49,50]. This is partly due to their lower BMI [41], but in our investigation, vegetarians and non-vegetarians were matched by BMI, and thus, we did not find any significant difference in those parameters.

In a recent meta-analysis, the prescription of a vegan or lacto-ovo-vegetarian diet was associated with a mean weight reduction of about 3.4–4.6 kg despite the absence of specific guidance on energy intake or exercise [51]. Therefore, our study, together with this recent investigation, underlines the need to encourage people to follow a plant-based diet.

In conclusion, a higher REE was found in vegetarians than in non-vegetarians, and vegetable fats in the diet may be a key factor for increasing it.

## 5. Conclusions 

Some macronutrients in the vegetarian diet, particularly vegetal fats intake, could have a role in increasing REE. Furthermore, vegetarian diet could change the cytokines profile which, in turn, could influence REE. However, prospective studies are needed to recommend this type of dietary intervention in the general population.
